# Supplementary material for: Evidence for an internal model of friction when controlling kinetic energy at impact to slide an object along a surface toward a target
Source: PLoS One. 2022 Feb 24;17(2):e0264370. doi: 10.1371/journal.pone.0264370 (PMC8870541; doi:10.1371/journal.pone.0264370)
Supplement: Attachment — Submitted filename: Response to Reviewers.docx [file pone.0264370.s001.docx]

Dear Reviewers,

First, let us thank you for your comments that helped us to improve greatly our manuscript. Please, find below our point-to-point response (in red color) to the comments.

**Reviewer #1:** The main claim of this study is that it has obtained experimental evidence that human motor control system uses internal model of friction. There is plenty of evidence for internal models of friction and clever strategies how they are used; regrettably the authors mention only a very limited set of studies.

**Response :** We added literature review about evidence for internal models of friction, in the *Introduction*.

The novelty in this study is claimed to be that it investigates ballistic movements.

Unfortunately the study design is flawed and there is absolutely no evidence of internal model of friction demonstrated in this study. By classical definition of "internal model" subject should be able to parameterize movements based on knowledge of friction. Ability to learn to perform movement when friction changes, is not an evidence of internal model of friction.

In this study the subjects' task was to hit a plastic cube-like object for it to slide to a given target distance. The subjects learned to perform this task minimizing the distance error. Then the same task was performed during 10 trials when surface was sloped at 10 degrees followed by another 10 trials when surface was sloped by 10 degrees in the opposite direction. After that the surface material and thus friction was changed and the procedure repeated. Again the subjects learned to hit the cube with appropriate speed and transfer kinetic energy (KE) to make it slide closer to the target. I see no discovery in the fact that people seeing under/over-shoot in the target distance can figure out that they have to hit stronger or weaker. For this to happen no model of friction is needed.

**Response :** Our rationale is now better explained at the very beginning of the *Results* section : “In the present study we assume that participants have an accurate internal model of gravity (as a physical invariant) and an accurate perception of surface slope. Therefore, if participants have a pretty good internal model of physics that links the initial cube speed to the final spatial error, spatial error should directly reflect participants’ internal model of µ (subjective µ). A subjective µ consistent with the physical µ would lead to no spatial error if participants are able to regulate their movement in order to impart the appropriate cube speed.” [...] Additional analyses were conducted on performance after a change in block condition in order to test if subjective µ generalized from one block to another with the same surface material. The rationale is that if participants reached an optimal performance (spatial error not significantly different from 0) at the end of a block, this would reflect the fact that they converged to the correct value of friction within a block. Accordingly, we theorized that participants should be able to parameterize their movement in spite of a change in block condition, for example a change of surface slope or target distance, in order to reach an optimal performance right from the first trial of the new block condition. If not, we expected signs of motor adaptation in the new block of trials suggesting that a recalibration of subjective µ across trials was needed”.

Regarding the point made about “Ability to learn to perform movement when friction changes, is not an evidence of internal model of friction.” We now explain in the Discussion section: “Although we agree with Hadjiosif et al. (2021) that motor adaptation within block does not require the updating of an internal model, our results provide some evidence that when a physical property is changed between blocks, such as surface slope, participants can appropriately re-parameterize their movement in order to directly reach optimal performance. This adapted behavior cannot be explained without invoking an internal model of physics, or luck ! On the one hand, the fact that sometimes performance is not optimal after block change suggests that updating a predictive forward model for movement control is run in parallel with, and independent of, the mechanisms underlying error-based movement adjustment across trials of a given block (see the so-called “direct policy learning” of Hadjiosif et al., 2021). On the other hand, participants sometimes directly showed optimal performance at the first trial of a block in spite of a dramatic change in physical properties such as surface slope, surface material, and target distance simultaneously (see Figure 7). This suggests that the adequate change in cube initial speed at the initial trial of this new block resulted from a predictive forward model-based movement re-parameterization. This was possible because participants tuned their subjective µ of the surface material during previous blocks with a different target distance (see Table 1, the first three blocks with the same surface).

The only case for unsuccessful movement re-parameterization when three physical properties changed between blocks corresponded to the more demanding transition (see Figure 7) that required an increase of about 120% of the initial cube speed (see Table 2, from 0.967 m/s for a 25 cm target distance on a -10° aluminum surface, to 2.147 m/s for a 50 cm target distance on a 0° balsa wood surface). Participants undershot the target distance. If one considers that predictive forward models are encapsulated knowledge of the physical properties of the environment and of the motor system (see Miall et al 1996), underestimation may reflect the influence of an explicit/conscious knowledge on motor control. This explicit influence may correspond to an adaptive prior knowledge according to which underestimation is relevant for survival. Along the same lines, Joh et al (2007) showed that underestimating high friction slopes permitted participants to attempt standing on steep slopes without risk of slipping.”

Put it this way – if there would be no internal model of friction, what would the result be then?

**Response :** Without an internal model of friction we expect motor adaptation within each block of trials.

Even with this flawed experimental design one might attempt to extract some hints of possible internal model of friction existence, if learning speed would be compared between the first block of conditions and the second when surface friction was changed. No such analyses was attempted and data were analysed regardless whether given frictional condition was present in the first or in the second block.

**Response :** We thank the reviewer for suggesting these analyses that we ran and correspond now to Figure 5, 6, and 7. They provide more substantial evidence in favor of the “possible internal model of friction existence”.

The conclusion that "internal model of friction can be generalized when the slope changes" is simply wrong and the logic is upside down what the experimental design allows to conclude - the friction was not changed and remained the same when blocks with different slope angles were tested. How authors arrived at such conclusion is absolutely not clear. If anything, this study reconfirms that there might be an internal model for slope angle, but absolutely no evidence for internal model of friction.

**Response :** The reviewer misunderstood our rationale. Indeed, our rationale is the other way round with respect to the reviewer's logic, as we explain at the beginning of the Results section: “In the present study we assume that participants have an accurate internal model of gravity (as a physical invariant) and an accurate perception of surface slope. Therefore, if participants have a pretty good internal model of physics that links the initial cube speed to the final spatial error, spatial error should directly reflect participants’ internal model of µ (subjective µ). A subjective µ consistent with the physical µ would lead to no spatial error if participants are able to regulate their movement in order to impart the appropriate cube speed.”

The friction coefficient µ is indeed associated with a given surface material. Reviewer 1 sentence: “the friction was not changed and remained the same when blocks with different slope angles were tested” only holds for blocks with similar surface material. There were several instances of block transition with surface material change (see the new Table 1) which required a change in the subjective µ according to our predictive forward model-based movement re-parameterization approach.

For example, the simplest experimental design, which actually would be able to demonstrate internal model of friction, might be that the subjects would learn to slide the same cube within two channels, lined with two surfaces with different frictional properties. Then another cube with a different mass would be introduced. Subject would learn to slide this cube to hit the target distance within one of the channels with one of surfaces. Then taking this cube to a second channel with a different, but known friction and repeating the task. If there would be internal model of friction then subjects would be able to apply the friction model to adjust KE to the new frictional condition. This would be evidence of frictional model as no explicit learning of KE would be possible as no such condition has been encountered before. Thus it would indicate that the required KE must have been estimated (modeled) based on internal model of friction coefficient.

**Response** : Reviewer 1 suggests that manipulating object mass would provide more definitive evidence in favor of an internal model of µ (friction coefficient) for the control of object KE. As illustrated in the equation below (that we now included at the end of the *Discussion* section), It is true that the required object KE to send it toward a given target distance (*d*) increases with object mass. Keeping surface slope (𝛂) constant while varying object mass changes the required object KE.

However, one should keep in mind that object mass has no impact on optimal object speed (see the left part of the equation). In terms of perception-action control, our contention is that participants used a visual-based (on the basis of v_0_ and spatial error) control of movement rather than a proprioceptive-based control (on the basis of the reaction forces from finger-cube contact). Indeed, proprioception of hand impact seems penetrable by visual motion input as shown by Arai & Okajima (2009 <https://doi.org/10.1167/9.11.19>) and Watanabe (2013 <https://doi.org/10.3169/mta.1.199>). Moreover, we now indicate at the end of the “Motor coordination efficiency” section that “although Total KE predicts linearly Cube KE (wrist strategy *R*² = 0.92 ; forearm strategy *R*² = 0.96), the slope of cube KE as a function of Total KE is far from 1 (Slope_wrist strategy_ = 0.075 ; Slope_forearm strategy_ = 0.097), reflecting the fact that KE efficiency (Cube KE/Total KE) is only about 10%. The other 90% is conserved in the arm movement after contact, and in the deformation of the finger(tip) during the contact.” The later results question a proprioceptive-based control (on the basis of the reaction forces from finger-cube contact) of movement.

Yet, according to the equation below, optimal initial cube speed (v_0_) varies with surface slope (𝛂), but not with object mass. This is the reason why we chose to manipulate surface slope (𝛂) rather than object mass. The experiment suggested by the reviewer builds on the fact that object mass is a parameter of object KE that must overcome the resistive force in order to send the object to a given target distance. However, according to Equation 10, if a participant doesn’t succeed in sending the object of another mass to a given target distance on a new surface material, we wouldn’t be able to isolate the source of the error: whether in the estimation of µ or of the object mass.

Still, we included a new paragraph in the *Discussion* section suggesting how manipulation of object mass may be helpful in future experiments : “Further investigation of the role of visual perception of the initial object cube speed for the control of movement in our task may require experimental manipulation of both visual input and object mass. As illustrated in equation 10 object mass has no impact on optimal object speed to reach a given target distance (see the left part of the equation). What is important is the initial object cube speed irrespectively of its mass. Let’s take an example with a cube sliding on a horizontal surface whereby the participant performs a block of trials on an aluminum surface, and then another block on a balsa wood surface. Assuming that the subjective µ value of each surface has been correctly tuned at the end of each block, then if the participant is given a cube of different mass (a preliminary psychophysical experiment may be needed to estimate participant’s differential threshold for object mass) we expect that she/he will send the new cube to the target distance accurately, by imparting the appropriate cube KE in order to get the same initial cube speed as before for each surface material. In contrast, if the first half of the target distance was occluded in the initial two blocks, and therefore the initial cube speed was never available visually, we expect substantial spatial error after the change in cube mass.”

The second largest section of the manuscript deals with multiple strategies how subjects regulated KE of the hit in this constrained experimental situation. I don’t see how it is directly relevant to the question about internal model of friction, thus I see no value in it, but I hope this information could be useful for someone. The authors would need to explain better what it reveals what is important beyond specifics of this manuscript.

**Response** : We rephrased the end of the *Introduction* section in order to better explain why we investigated motor coordination (namely for testing motor redundancy when performing the task) : “Moreover, we tested the assumption that motor redundancies [24,25] would allow for variability in motor coordination to reach the required kinetic energy at impact.”

Minor

The directory address, where the authors claim the study data are made available, was empty at the time of review.

**Response** : We provide a new link to a directory containing the data, together with matlab processing codes, and an explanatory “README FIRST MATLAB program.pdf” file.

-------------------------------------------------------------------------------------------------------------------------------

**Reviewer #2:** General: The manuscript by Famie and colleagues reports a study which aimed to provide evidence for the existence of a context-specific representation of the friction of a surface, over which an object had to be pushed. Participants were instructed to slide with a hitting motion an object across surfaces of varying friction (aluminum vs. balsa wood) at a specific target distance (25 vs. 50 cm) under different conditions of surface slope (0, 10, -10 degrees). For each task condition, performance curves were plotted for the spatial error of reaching the target distance and the initial speed of the object after it was hit. The experimental protocol was arranged in such a manner that participants were first exposed to the horizontal surface to acquire an estimate of the friction of each surface. Learning was demonstrated by fitting exponential performance curves across the ten trials of each condition. In terms of a transfer condition, participants than had to perform in the up- or downward sloped conditions, which did not express similar learning but nearly optimized performance instead. Based on the motion equation of the observed initial speeds, the participants’ subjective friction estimates were derived. The progression of the spatial error of reaching the required distance was considered the indication of the acquisition of the internal model of a surface’s friction. In addition, participants’ arm and wrist kinematics during the striking motion were analysed to determine their individual strategies for regulating to invested amount of kinetic energy. Two groups of participants could be established: one group that only used a wrist motion and one group that used an elbow and wrist motion.

The study described in this manuscript made use of a very sophisticated methodology. I appreciate its scientific rigor and interesting conclusions. I follow the authors’ arguments but the only aspect that I miss in the discussion of the manuscript is an integrated perspective, which combines an internal model of friction with an internal model of gravity. Do the authors believe that a representation of friction is the only factor that influences performance in the two conditions, where the surface shows a downward or upward slope, or how are the two representations combined? Perhaps the author can elaborate this a bit more.

**Response** : We added the following sentences in the *Discussion* in order to better explain how an internal model of friction may be combined with an internal model of gravity: “Of course, our reasoning builds on the assumption that we have a “pretty good” internal model of gravity [23]. If participants' internal model of gravity is wrong, then the brain would need to solve equation 9 with two unknown factors, namely µ and g. However, motor control would still be possible provided that the subjective g value is a constant. If the subjective g value is greater than 9.81 m/s² then the subjective µ would be smaller than the actual µ value. The brain would only need to apply a scale value to the subjective µ in order to parameterize the movement so that it provides the required initial cube speed.”

Minor:

Page 3, paragraph 1: “Amorim […] where an object […] should stop after being launched due to a collision (with a launching object)”. I find this summary of the Amorim et al. study unclear, perhaps it can be clarified better.

**Response** : We rephrased the summary of the Amorim et al. (2015) study.

Page 9, equation 3: the equation lacks a squared bracket

**Response** : We corrected this typo. Thank you.

Page 9, table 1: I find the ordering of the table counterintuitive

**Response** : We changed the ordering as requested, in order to make the table more readable.

Page 11, paragraph 2: “We used […]”. This sentence is redundant given the first sentence of the first paragraph of page 10.

**Response** : We deleted this redundant sentence, as suggested.

Page 11, paragraph 3: “Motor adaptation […]”. Where exponential curves fitted for each individual participant or just for the group averages? I think the latter would be a weakness as it is unclear how well the function fits represent the individual participants performance curves.

**Response** : The fits illustrated in Figure 3 and 4 were computed at the group level. However, in order to get a better idea of the interindividual variability for the exponential curves, we provide below a figure of the Motor adaptation trend *R*² values as a function of the predicted spatial error at trial 1 (left figure) and of the predicted initial cube speed (m/s) at trial 1 (right figure), according to the exponential curve of each participant. As can be seen, there is a large interindividual variability in *R*² values (Y axis), which is the main reason why we chose to show the exponential curves fitted for the group averages. On the one hand, the linear fits below illustrate the fact that the exponential trend better explains individual data when the data at initial trial is farther away to optimal performance, and therefore adaptation is needed. On the other hand, the fact that for a substantial amount of data the *R*² values of exponential curves are smaller than 0.5 suggests that the data is noisy at the individual level, and therefore group averaging is necessary to improve the signal-to-noise ratio of the exponential performance change across trials. Note that we excluded from the data analysis (illustrated below) the individual data points for exponential curves with *R*² < 0.10 (about 1/5 of the trials), because it made no sense to consider predicted values from an unreliable model.

Page 14, paragraph 2: “The ANOVA also showed […]”. Which ANOVA? Perhaps here it could be a bit more specific.

**Response** : We performed “repeated measures ANOVAs” as indicated in the last sentence of the first paragraph in the *Results* section : “Repeated measures ANOVAs on the performance variables were performed with the following within-subjects factors: two target distances (25 cm and 50 cm) x two surface materials (balsa wood and aluminum) x three surface slopes (-10°, 0° and +10°) x ten trials.”

In order to refresh the reader’s memory, we added again the “repeated measures” information in the sentence pointed by Reviewer 2.

Page 15, paragraph 1: This description of Figure 4 does not seem to follow the order of the figure panels. Perhaps the description could refer to the figure panels better.

**Response** : The description of Figure 4 starts before the paragraph that Reviewer 2 is referring to, describing the results for the right panels (balsa wood), and starting with “For Balsa wood, motor adaptation was necessary for both target distances, due to an initial underestimation of friction. As a result, “. Indeed, the beginning of the FIgure 4 description starts in the previous paragraph starting with : “The repeated measures ANOVA also showed a significant interaction between Surface material, Target distance and Trial, F(9, 288) = 1.93, p = 0.048, for spatial error (see Fig 4). These results suggest a more efficient re-parameterization of the striking movement for Aluminum,”. Unfortunately, we positioned the legend between both paragraphs. Therefore, in order to avoid confusing the reader, we now moved the Figure 4 placement before the paragraph starting to describe Figure 4 results.

-------------------------------------------------------------------------------------------------------------------------------

**Reviewer #3**: Review of PONE-D-2108226

Summary

The authors examine whether humans have and update an internal model of friction. To this end, they asked participants to slide objects of different materials to two different distances along a path that had one of three different inclinations. By presenting conditions in a blocked manner, the authors could examine whether motor behavior developed over trials on the basis of the observed error between the required target position and the cube’s final position. The results show that participants adjusted their behavior over the trials to foster proper task execution, but only in some conditions. Despite the adjustments in some of these conditions, spatial error in the last trial was sometimes pronounced. These suggest that humans can utilize the observed outcome of a performed trial to update the motor plan of the upcoming trial, however this motor updating seems to occur under specific circumstances and appears incomplete.

First comments

This novel study will be of interest for the scientific community, especially those disciplines related to the planning and control of human movement and the role of physical properties, such as friction, in this. The main claims are incorporated within appropriate theoretical frameworks. The analyses are generally suitable. The results support some of the claims, though I have some suggestions below for improvements in this aspect. For instance, a general calibration of the internal model of friction, as implied in the abstract, cannot be concluded based on the data. Generally, the presentation of the results, and as a consequence the appreciation of the conclusions, could be more comprehensible.

The data and experimental code are not available right now. The authors provide a link where some material can be found, but the linked folder is empty.

**Response** : Sorry about that. Data and code are now fully available from the new link, together with an explanatory “README FIRST MATLAB program.pdf” file.

Below I provide detailed comments on what I believe could be improved in the manuscript. I hope that the comments can be helpful to the authors to further improve their nice work.

Comments

One of the main claims is that an internal model of friction can be generalized when the slope of the sliding surface changes. This is a rather central claim, considering that it is also highlighted in the abstract of the study (lines 27-28). However, based on the results, this claim seems inappropriate. In fact, participants’ behavior for both objects is stably suboptimal across trials when the sliding surface is at -10 deg, as we can see in Figure 3 (upper left panel).

**Response** : We do not understand why the Reviewer 3 finds performance “stably suboptimal” for the -10° (upper left panel) condition in Figure 3. Actually, participants reached optimal performance at the last trial of -10° (post-hoc tests showed that mean performance for both surface materials at trial 1 for -10° condition did not differ from zero). Moreover, there is no evidence of motor adaptation in the -10° condition, contrary to the initial 0° condition. As mentioned in the manuscript these findings are suggestive of generalization of an internal model of friction, namely the subjective µ value tuned at the initial 0° condition is generalized in the internal model of physics for the -10° condition.

Similarly, when the surface is at +10 deg, performance for the wooden object also does not improve across trials.

**Response** : First, regarding “the wooden object” in Reviewer 3 sentence, we would like to draw the attention of the reviewer to the fact that the surface material was either in balsa wood or in aluminum, and the cube object was always in PLA (polylactide) (see the “Experimental setup” sub-section). Still, we guess that the reviewer is referring to the balsa wood data points (black dots) in Figure 3 upper right panel (+10° condition). If so, we do not understand Reviewer 3 interpretation of the data because factually our data show that performance (as measured by spatial error) at trial 1 and trial 10 on balsa wood did not differ statistically from zero (optimal performance). Descriptively, these mean data points (black dots) show a fluctuation in performance across trials without any signature of motor adaptation, but let us insist on the fact that, in spite of this fluctuation, performance (as measured by spatial error) at trial 1 and trial 10 on balsa wood was statistically optimal (that is, not different from zero).

These suggest that, even though participants can see that their actions did not bring the object at the correct place, they do not tailor their motor execution to the appropriate extent in order to overcome this error. As a matter of fact, the initial cube speed (Figure 3 – lower panels) is rather constant in the above-mentioned conditions.

**Response** : Again, we do not understand the point that Reviewer 3 is making regarding “rather constant” initial cube speed in the above-mentioned conditions. There is no reason to expect motor adaptation in initial cube speed for the conditions showing no adaptation in spatial error, namely both surface materials at -10°, and balsa wood at +10°. Spatial error data points show that performance was close to optimal (that is, not different from 0% spatial error) in these conditions, and precisely for this reason, initial cube speed did not show motor adaptation, simply because motor adaptation was not needed.

In contrast to this suboptimal behavior, participants do adjust their movements when the sliding surface is at 0 deg, for both objects, as well as for the aluminum-object when the sliding surface is at +10 deg. Nevertheless, such motor adjustments can be incomplete (wooden object at +10 deg),

**Response** : First, let us specify again that the object was always the same PLA cube-like object. Also, we do not agree with the “suboptimal” interpretation (see our Responses before), we agree that participants went through motor adaptation in the initial 0° condition. Our interpretation for this motor adaptation in the 0° condition is straightforward : the 0° condition was the first condition with the surface material that participants underwent, and therefore motor adaptation reflected somehow (but not only) the tuning of the subjective µ for each surface material.

Regarding the motor adaptation for the aluminum surface at +10°, the “big picture” is a little more complex than Figure 3 would suggest. Indeed, when looking at the new Figures 5 and 6 we can see that there was no evidence of motor adaptation for the Alum_+10° condition when it followed a 0° slope for the 25 cm target distance (see Figure 6 upper left panels). Therefore, the presence of motor adaptation for the Alum_+10° condition in Figure 3 varied with the slope and target distance condition in the preceding block: such adaptation was observed when Alum_+10° followed 0°_50cm (Figure 6), or -10°_25cm and -10°_50cm (Figure 5).

So the updating of an internal model may either be partial or the observed errors may arise from other sources than only the internal model (e.g., motor noise). These issues may deserve some more (explicit) discussion. In any case, I think it is important to avoid generalizations when it comes to the adaptation of the internal model.

**Response** : We fully agree with Reviewer 3 about the role of motor noise and modified our *Discussion* accordingly, see “Likewise, along the lines of Sternad (2018) we suggest that variability in spatial error across trials, and of KE as a function of optimal cube speed, may reflect random exploration of motor strategies in order to achieve the task. The source of this “motor noise” is intentional (for the purpose of reducing spatial error), contrary to physiological noise in neural control signals (Harris & Wolpert, 1998) that may add variance in performance too. Participants intentionally learn to control various sources of noise in performance such as KE efficiency (the loss of KE between Total KE and Cube KE), the orientation of cube trajectory after impact (to avoid or to exploit collisions between the cube and the gutter), etc. These different sources of noise may also account for the partial generalization of subjective µ between blocks of trial.”

A question that arises is why the adjustments are observed mainly in the 0 deg condition. It might be the case that participants have difficulties to estimate the role of gravity, which does not necessarily interact with friction. This too seems to deserve a more clear discussion.

**Response** : As Reviewer 2 described “The experimental protocol was arranged in such a manner that participants were first exposed to the horizontal surface to acquire an estimate of the friction of each surface.” Accordingly, we theorized that motor adaptation in the 0° condition reflected the tuning of the subjective µ value. In order to comply with Reviewer 3 comment (as well as the other two reviewers) about the estimation of g and µ, we added the following sentences in the *Discussion* : “Of course, our reasoning builds on the assumption that we have a “pretty good” internal model of gravity [23]. If participants' internal model of gravity is wrong, then the brain would need to solve equation 9 with two unknown factors, namely *µ* and *g*. However, motor control would still be possible provided that the subjective *g* value is a constant. If the subjective *g* value is greater than 9.81 m/s² then the subjective *µ* would be smaller than the actual *µ* value. The brain would only need to apply a scale value to the subjective µ in order to parameterize the movement so that it provides the required initial cube speed.”

Equation in line 183: It would be helpful to mention what are the variables in the equation (e.g., what is alpha?). Similarly for the equations in lines 223-224 (what is L?). Apologies if these are mentioned and I did not notice.

**Response** : We now define surface slope (α) in the sentence following equation (2); thank you for mentioning this point. Regarding L, it is defined in the sentence just after equation (6): “In equations (4) and (5), the segment lengths (L) were reported following the ISB recommendation [28]”.

Equation in lines 223-224: There is a mistake in the bracketing, please adjust accordingly.

**Response** : Sorry for these bracketing typos, that we corrected in the revised manuscript.

Equation in lines 223-224: Is it indeed true that the length of wrist is multiplied with the length of the elbow as seems to be the case in the end of line 223?

**Response** : Oops, thank you very much. We now corrected this typo (that occurred in the text, not in our matlab code !): a “+” sign was missing in the manuscript.

The presentation of the results sometimes becomes too technical and it is hard to get the main message. This is a general comment but particularly for lines 376-405. I recommend adding some higher-order information to guide the reader along the results and to highlight what do the reported effects mean.

**Response** : This sentence in the original (and revised) manuscript, just before the lines Reviewer 3 is referring to, already provided higher-order information: “Similar to Fig 8, Fig 9 shows an equivalent recruitment of the wrist and forearm in the Forearm strategy and a much greater recruitment of the wrist in the Wrist strategy.” However, in order to facilitate the interpretation of the subsequent analysis, we added more higher-information along the results.

Line 291: Why is the first trial of the +10 deg compared against the last trial of the 0 deg condition, and not with the last trial of the -10 deg condition, which is the condition presented just before. This way, the authors can examine whether the performance in trial_1 of +10 deg condition is similar to the most recently performed trial of the -10 deg condition.

The answer to this may be the explanation that is given in lines 259-261, however, in this case, this information is somewhat lost until the results are presented. Therefore, if this is the answer to my question, I suggest reminding the reader about the purpose and meaning of the comparison by giving this higher-order information at the respective Results-sentence. In any case, I am not fully convinced about the relevancy of this comparison (i.e. first trial in +/- 10 deg condition against the last trial of the 0 deg condition). If the purpose of the study is to examine whether participants tuned their internal model across repetitions of the same condition, then the authors should look whether participants learned from their error during the 10 trials of that conditions. What is the contribution to the research question of the comparison between trial_1 in +/- 10 deg and trial_10 of 0 deg condition? It just adds more statistical tests, so it makes it more likely to obtain a statistically significant result, which may in fact be random. Therefore, I would be tempted to suggest to remove any comparisons of the first trial in +/- 10 deg condition with the last trial of the 0 deg condition -unless it becomes explicit that such comparisons are central to the purpose of the study. Removing these would also help focus the results in the main, central findings, contributing to a more concise Results section.

**Response** : Thank you for these comments and suggestions that led us to add new analyses regarding the performance change between blocks, by taking into account the different block orders of our groups. We hope that these new data and resulting discussions will bring additional evidence toward the tuning of a subjective µ value for the parameterization of movement.

Lines 446-448: The reduced finger speed at the time of contact in the Forearm-strategy group may indeed be due to a tuning of motor behavior on the basis of the increased kinetic energy, compared to the Wrist-strategy group. However, when rotating the wrist, the finger is by definition also rotated, so it is not surprising that the Wrist-strategy group shows higher index finger speeds. Considering this, the sentence in these lines may be somewhat misleading and it may require some rephrasing to also account for the above-mentioned possibility.

**Response** : Index finger speed depends on both the rotation of the forearm and the rotation of the hand. The comment that “when rotating the wrist, the finger is by definition also rotated, so it is not surprising that the Wrist-strategy group shows higher index finger speeds.” only holds if both groups do not differ in terms of forearm rotation speed. However, as illustrated in the new Figure 8 (previously Figure 5) and 9 (previously Figure 6) our data show that both Motor strategy groups differ in forearm angular amplitude and speed.

Minor

It would be helpful to add p-values in all reported tests, even if these are non-significant. This is sometimes done (e.g., line 281), but some other times it is not (e.g., 267).

**Response** : We added the requested information in the revised manuscript.

Line 265: “significant spatial error”. What is the comparison that is being made here for the first trial “regardless of material”? Is it trial_1 vs some other trial? Or is it trial_1 between different orientations of the surfaces? Also, in line 266 there is a t-test result, but it is not clear what comparison this test refers to.

**Response** : We rephrased the results as follows: “The results showed significant spatial error (as compared to zero) in the initial trial regardless of the surface material (as illustrated by the asterisks in Figure 3, for Bonferroni corrected paired *t* tests). Moreover, spatial error was significantly greater for Balsa wood (*M* = -29.57%) compared to Aluminum (*M* = -12.66% ), *t*(32)=3.40, *p* = 0.002.”

Line 291: “increased”. This reads as if there is an effect, however the statistical comparison does not support this wording. I recommend rephrasing. Line 292: “in order to”…this reads as if participants aimed to achieve a spatial error. Consider rephrasing to something like “resulting in a similar spatial error…”.

**Response** : We rephrased these results.

Line 293: “was required”…so for the wooden object there was no requirement for motor adaptation? It is clear that already from the first trial there were spatial errors, and thus performance suboptimal. So, I would say, motor adaptation was required for both objects, but it was achieved only for the aluminum one. Still, the adaptation achieved in the aluminum object seems to be suboptimal as the spatial error appears quite strong in the last trial.

**Response** : Along the lines of Reviewer 3 comment, we rephrased the description of the results by referring to suboptimal adaptation for the Aluminum surface (N.B.: the object was always in plastic, only the surface material changed). However, we rather disagree with the “overall suboptimal performance” interpretation of the results for balsa wood in the 10° surface slope condition, because it is not supported by our statistics showing that performance did not differ from zero, neither at trial 1 nor at trial 10 according to our Bonferroni corrected *t* tests.

Lines 353-354: Is the “forearm” strategy correctly named? The authors state (and the figures show) that in this ‘group’, participants used both the forearm and the wrist. Referring only to the forearm as a ‘strategy’ may give a wrong impression. An idea would be to use a more vague term, such as ‘hybrid’ strategy. However, this is truly a minor comment, as the authors explicitly state that the forearm strategy involves both the wrist and the forearm.

**Response** : We chose to use the “forearm strategy” terminology although it involves both the wrist and the forearm, in order to put forward the major source of difference in the coordinations, namely, a greater forearm rotation in the “forearm strategy”.

Lines 434: Is there a reason that the factor ‘slope’ is not considered here?

**Response** : Neither “Surface slope” factor (3 levels) nor the “Target distance” factor (2 levels) are directly considered in the KE analyses because they are already included in the 6 data points of the optimal cube speed variable, as explained in Table 2.
