# Supplementary material for: Evidence for an internal model of friction when controlling kinetic energy at impact to slide an object along a surface toward a target
Source: PLoS One. 2022 Feb 24;17(2):e0264370. doi: 10.1371/journal.pone.0264370 (PMC8870541; doi:10.1371/journal.pone.0264370)
Supplement: Attachment — Submitted filename: Response to Reviewers.docx [file pone.0264370.s002.docx]

**Reviewer #1:**

Thank you for addressing my comments. I don't fully follow the logic of the authors, but based on thorough data analyses performed I want to believe that you have well thought this through and I respect your opinion. Probably the same question could have been addressed in more straight forward experiment requiring less convoluted analyses. I am not sure who will benefit from this work and unnatural experimental task, but I hope someone will. The most relevant studies in the context of meaningful and practical motor tasks, like dexterous object manipulation, are still not even mentioned. Such studies have plenty of evidence for internal models of friction and clever strategies how they are used.

**Response :** We now refer to dexterous manipulation in the Introduction, as suggested : “Finally, the literature on dexterous manipulation suggests that the appropriate control of grip force may be informed initially by adapting internal models built from previous experience in various contexts, and flexibly adjusted on the basis of sensory feedback. [13-14]”

**Reviewer #2:**

The authors have addressed all recommendations to my satisfaction. They closed gaps in their presentation by the inclusion of additional results and extension of the discussion section.

**Reviewer #3:**

I thank the authors for their responses to the previous comments. I still believe that the results are of interest, however I am quite lost in the messages that the authors aim at conveying. The additional revisions have improved some parts but made some others either bulky or technical, while information about the analysis procedures are obscured or rather unclear. Below I would like to start first with some clarifications from my side on my previous comments. Then I provide my comments on the revised manuscript, as well as suggestions for improvements on the presentation and interpretation of the work.

**Clarifications of some previous comments** The authors in their response letter state that they do not understand why I consider performance ‘stably suboptimal’ in some conditions. In particular, Figure 3, upper left panel, shows spatial error in the -10 deg condition. Here we see that the presented averages for each trial and material do not follow any consistent pattern. To be more precise, hoping that this will make clearer what I mean, for the Aluminium surface, the cube overshoots the target by ~8%, then a ‘correction’ occurs (trial 2) followed by a sequence of gradual undershoots (trials 3-5). Then participants overshoot again, before minimizing the error in the last 3 trials.

When it comes to the wooden surface, again in the same panel, participants systematically undershoot, sometimes more, sometimes less, until the last 3-4 trials, when their spatial error is around zero. These results, particularly those for the Aluminium surface, show that performance is stably changing across trials, but that this change is not systematically in one direction (as, for instance, in the 0-deg condition). This does not necessarily mean that people do not learn, of course. The lack of an exponential fit may reflect an exploratory strategy, during which subjects try out different ways of hitting the object until they find the one that works ‘best’.

**Response :** Thank you for these clarifications and the “exploratory strategy” idea that we now use in the Results when interpreting the “Mean performance and motor adaptation” data.

Alternatively, the use of the spatial error by the authors may be incomplete in capturing performance. Right now, the authors consider the error as the distance between the front part of the cube and the ‘target’s distance’ (page 11, lines 222-225). Considering that the triangular sticker and the respective marker are also not clearly evident in Figure 1, as the authors refer to that figure, I cannot tell whether it is possible that participants could exploit a range of possible endpoints that can be considered an accurate end-position. If this is the case, then the authors could consider highlighting on the graph which % range belongs to any part of the target area matching the front end of the cube.

**Response :** The position of each target sticker was non-ambiguous, and the participants clearly understood the instructions (aligning the front part of the cube and the ‘target’s distance’), as most of the last trials show. We now added a zoom in Figure 1 to highlight a sticker target distance.

In my previous review, I mentioned that performance for the wooden condition in the +10 slope does not improve over trials. At least seeing from the graph, I insist on that and cannot understand the authors’ response. As the authors also state, spatial error is similar between trials 1 and 10, and I guess between all other trials in that condition (wooden, +10). So, I guess they actually agree with my comment, namely that performance did *not* change across trials.

**Response :** Thank you for providing this clarification with which we agree. Our data show that performance (as measured by spatial error) at trial 1 and trial 10 for balsa wood did not differ statistically from zero (optimal performance), as you mentioned. Descriptively, these mean data points (black dots) show a fluctuation in performance across trials without any signature of motor adaptation. In spite of this fluctuation, performance (as measured by spatial error) at trial 1 and trial 10 for balsa wood was statistically optimal (that is, not different from zero, with Bonferroni correction).

**Comments**

As suggested before, the authors now provide more p-values, but not in all instances. There are several places where they report that the p-value is below the alpha level (e.g., page 14, line 277). Although it is important to remind the reader about the alpha level, the p-value is not given. The p-values need to be provided explicitly (and the alpha-level can be provided as a reminder whenever necessary).

**Response :** The “ (e.g., page 14, line 277)” example that is being referred to belongs to Figure 3 caption last sentence : “The Asterisks for spatial error (mean ± SE) indicate initial and final means differing significantly (Bonferroni correction with p < 0.05/12 = 0.004) from zero.” In order to simplify the text reading, we initially chose to focus on the statistical criterion when performing Bonferroni corrections, instead of providing the exact *p* value. We now provide both as requested, in the revised version.

However, as indicated in PLOS ONE guidelines, *p* values less than 0.001 were expressed as *p* < 0.001 (<https://journals.plos.org/plosone/s/submission-guidelines#loc-manuscript-organization>) : “**P-values.** Report exact p-values for all values greater than or equal to 0.001. P-values less than 0.001 may be expressed as *p* < 0.001, or as exponentials in studies of genetic associations.”

Following this, I would like to emphasize that the authors chose to sometimes correct for 12 comparisons, setting the alpha-level to 0.004. This is good when it comes to type 1 errors but it also shows that the design chosen by the authors together with the Bonferroni correction is conservative. Considering these, authors need to be more transparent about their procedures and results. Namely, a detailed overview of the statistical procedures should be provided in the Methods (not in Results), explaining *when* do they correct and for how many multiple comparisons.

**Response :** In the new revised version, we now moved these details about statistical procedures in the *Performance variables* subsection of the *Methods*, instead of the *Results*, as requested.

Right now, there are some explanations spread across the Results that makes it difficult to appreciate the procedure, focus on the findings, and appreciate the interpretations. In addition, **reports of whether the data are normally distributed or not should be added, so that the applied statistical tests can be justified.**

**Response :** The distributions of our data look descriptively normal. Let’s exemplify this normality issue with the distributions for the manuscript Figure 3 individual data (see below). Although the one-sample Kolmogorov-Smirnov test indicates that the distributions are normal, the more stringent Shapiro Wilk test suggests that the data are not significantly normal. We tried different transformations (log, sqrt, etc) that did not improve normality of Shapiro Wilk test criteria (it even worsened the test result, by inducing changes in the distribution shape when in fact our data were already very close to normality).

Note that, as recognized in statistics handbooks, the tests of whether a distribution is normal (the Kolmorgorov-Smirnoff and Shapiro-Wilk tests) “are based on null hypothesis significance testing and this means that (1) in large samples they can be significant even for small and unimportant effects, and (2) in small samples they will lack power to detect violations of.” (Field, 2016, see below). Significance for small and unimportant deviation from perfect theoretical normality is exactly our case.

In fact, **the Q-Q plots** show that our data appear to be normally-distributed with most of the individual trials on the diagonal. Note that only simulated data would give perfect diagonal fit. It is true that there are a few outliers, especially for spatial error, that we decided not to suppress. These are values from the -10° condition where participants overestimated the required initial cube speed. In the -10° slope & 25 cm target distance & aluminum surface condition, an initial cube speed too high would bring the object close to the end of the gutter and lead to a 150% (37 cm overshoot, bringing the object at 62 cm from its initial position) or even 200% spatial error (50 cm cm overshoot, bringing the object at 75 cm from its initial position, that is close the gutter end). These extreme errors happened but only a very few trials (less than 10) out of the 1980 trials illustrated in the Q-Q plots.

Moreover, it is well-established that ANOVA (this holds also for *t* tests, because comparing two means with ANOVA or *t* tests would give similar *p* values) is robust to violations of normality in terms of Type I error (see References below). As a consequence, given our very close to normal data, and in order not to enter into heavy justifications with respect to ANOVA and the normality assumptions, we added a sentence in the Results section: “Although ANOVA is robust with respect to violations of normality in terms of Type I error (Blanca et al., 2017), Q-Q plots showed that our performance variables data appear to be normally distributed.“

------------------------------------

According to Andy Field (2016, <https://www.discoveringstatistics.com/repository/exploringdata.pdf>) : “Many people take the ‘assumption of normality’ to mean that your data need to be normally distributed. [...] The misconception that people often have about the data themselves needing to be normally distributed probably stems from the fact that if the data are normally distributed then it’s reasonable to assume that the errors in the model and the sampling distribution are also (and remember, we don’t have direct access to the sampling distribution so we have to make educated guesses about its shape).” [...] “Throughout this handout we will look at various significance tests that have been devised to look at whether assumptions are violated. These include tests of whether a distribution is normal (the Kolmorgorov-Smirnoff and Shapiro-Wilk tests), tests of homogeneity of variances (Levene’s test), and tests of significance of skew and kurtosis. All of these tests are based on null hypothesis significance testing and this means that (1) in large samples they can be significant even for small and unimportant effects, and (2) in small samples they will lack power to detect violations of. [...] The central limit theorem means that as sample sizes get larger, the less the assumption of normality matters because the sampling distribution will be normal regardless of what our population (or indeed sample) data look like. So, the problem is that in large samples, where we don’t need to worry about normality, a test of normality is more likely to be significant, and therefore likely to make us worry about and correct for something that doesn’t need to be corrected or worried about. Conversely, in small samples, where we might want to worry about normality, a significance test won’t have the power to detect non-normality and so is likely to encourage us not to worry about something that we probably ought to. Therefore, the best advice is that if your sample is large then don’t use significance tests of normality, in fact don’t worry too much about normality at all**.** In small samples then pay attention if your significance tests are significant but resist being lulled into a false sense of security if they are not.

Marshall and Samuels (2017) : "One of the assumptions for most parametric tests to be reliable is that the data is approximately normally distributed. The normal distribution peaks in the middle and is symmetrical about the mean. Data does not need to be perfectly normally distributed for the tests to be reliable."

DOI:10.13140/RG.2.2.33314.94405

REFERENCES

Blanca MJ, Alarcón R, Arnau J, Bono R, Bendayan R. (2017). Non-normal data: Is ANOVA still a valid option? *Psicothema*. 29(4):552-557. doi: 10.7334/psicothema2016.383.

Glass, G.V., P.D. Peckham, and J.R. Sanders. (1972). Consequences of failure to meet assumptions underlying fixed effects analyses of variance and covariance. *Rev. Educ. Res*. 42: 237-288.

Harwell, M.R., E.N. Rubinstein, W.S. Hayes, and C.C. Olds. (1992). Summarizing Monte Carlo results in methodological research: the one- and two-factor fixed effects ANOVA cases. *J. Educ. Stat.* 17: 315-339.

Lix, L.M., J.C. Keselman, and H.J. Keselman. (1996). Consequences of assumption violations revisited: A quantitative review of alternatives to the one-way analysis of variance F test. *Rev. Educ. Res*. 66: 579-619.

This Q-Q plot for the Figure 3 **spatial error** individual data shows that the standardized residuals fit nicely along the diagonal suggesting that the data appear to be normally distributed.

This Q-Q plot for the Figure 3 **initial cube speed** individual data shows that the standardized residuals fit nicely along the diagonal suggesting that the data appear to be normally distributed.

And ideally, individual data points should be used for visualization, according to modern approaches about data transparency, which should assist the reader in understand the nature of the reported (no-) effects (see also a later comment).

**Response :** Regarding data transparency, we would like to remind the reviewer that we provided all our data in the link associated with our manuscript : <https://doi.org/10.5281/zenodo.5705206>

The data used for the statistical analyses are available in the *1_Table_Data_for_Stats* files (one in *mat* format, and the other in *xlsx* format) inside the *3_Data_for_Stats folder* of the *Data.zip* file. We cannot be more transparent given that we also provide the raw data and matlab code used to generate these statistical data files.

Regarding individual data points visualization, instead of displaying each individual data point, which would lead to unreadable figures, we showed the data variability using error bars.

I also have a question about the sample size. Was that determined on the basis of some power analysis or other procedure? If not, why is the sample size not even so that each of the [0, +10, -10 deg] and the [0, -10, +10 deg] groups have the same number of participants?

**Response :** We did not determine the participant number from power analysis due to missing reference data in the literature for comparable studies. In fact, we initially had 40 participants (20 in each group) performing the experiment, but during motion data analysis it happened that for 7 participants the maker positioned on the distal phalanx was not visible for the cameras just before the contact with the cube, leading to many missing values in several conditions. This problem was discovered after we could re-run additional participants early enough to avoid material availability problems and thereby several months of delay for data gathering in this PhD work. We therefore decided to analyze the data of the participants without missing data.

**Presentation of testing**

This remains quite confusing to me. I had to read some parts several times to understand what comparisons the authors make, and for what research questions.

For instance, in page 14, line 285, the authors refer to Figures 3 and 4 to explain the number of tests applied, but this implicit information is hard to digest. Further, in page 15, lines 315-317, there are tests between trial 1 of condition n and trial 10 of condition n-1, but there is no information about the difference of each of these trials from zero (a no-difference between these two trials does not mean that one or both of these are also no-different from zero).

**Response :** Given that we modified the structure of the Results as recommended (see below), this content from “page 15, lines 315-317” of the previous version is now deleted.

A possible way to improve these, and other several instances across the manuscript, is to work on the structure of the Methods and Results. As an example, the authors could explain in the Methods what tests are they planning to report and with what (parametric or non-parametric) statistical procedures, using separate paragraphs, one for each testing procedure.

**Response :** As previously mentioned, in the new revised version, we now moved these details regarding the rationale of the statistical procedures (among which the comparisons involving trial 1 and 10) in the *Performance variables* subsection of the *Methods* section, instead of the *Results* section, as requested earlier.

For instance, they could state something like: “We first wanted to test whether there were systematic spatial errors in the first trial of each condition. For this, we calculated spatial error in trial 1 of each participant and for each of the 12 conditions, and submitted these in a 3x2x2 ANOVA. Interactions were explored with further ANOVAs, and main effects with Bonferroni-corrected t-tests.”

**Response :** Here, the reviewer is proposing an analysis of spatial error for trial 1 with a 3 (slope) x 2 (surface material) x 2 (target distance) ANOVA. However, given that the 3 (slope) x 2 (surface material) x 2 (target distance) x 10 (trial) four-factor interaction was not at all significant, *F*(18,576) = 0.44, *p* = 0.98, we do not see any statistical justification for performing sub-ANOVAs. We now added this information about the later non-significant interaction in the new revision (sorry, for not providing this information before, and focusing on significant interactions instead).

Then, in another paragraph they could go on with something like: “We then wanted to test whether the spatial errors differed between the first and last trials of each block. Here, we examined with 12 paired two-sample t-tests, one per condition, whether the spatial error in trial 1 was different from that in trial 10….”

And then, in another paragraph: “In order to examine XXXX, we also compared spatial errors between the first trial of a given condition with the errors in the last trial of the previously presented condition. For this purpose, we conducted this and that test…”.

Of course, these are just suggestions. They may not capture completely what the authors intent to do but serve as ideas and the authors may find better ways in streamlining and explaining the procedure and testing they applied.

**Response :** We took into account all these suggestions for simplifying the data analysis exposure, starting with the ANOVA on spatial error in trial 1. We better explain the new rationale of the data analysis in the "Performance variables" section of "Materials and Methods".

**Results**

There is a good deal of the manuscript devoted to the results shown in Figure 3. Though this makes sense, the authors subsequently show that there are differences in performance between the [0, +10, -10 deg] and [0, -10, +10 deg] groups. In page 15, line 312, the authors remind the reader about the two groups. Yet, they continue with reporting the average performance. This is confusing because the first sentence of the paragraph makes the reader think that the following results (say, of the -10 deg condition) refer to when that condition was presented right after the 0-deg condition (which, apparently is not true, but this becomes clear only later). Please make more explicit in the Results that you first report average performance for each condition; a sub-section in the beginning of the Results may help in clarifying this.

**Response :** Now, we better explain the new rationale of the data analysis in the "Performance variables" section of "Materials and Methods", as suggested by the reviewer above : “As an example, the authors could explain in the Methods what tests are they planning to report and with what (parametric or non-parametric) statistical procedures, using separate paragraphs, one for each testing procedure“.

Also, please explain why Figure 3 does not show data separately for each (25 and 50 cm) distance.

**Response :** Figure 3 does not show data separately for each (25 and 50 cm) distance because the 3 (slope) x 2 (surface material) x 2 (target distance) x 10 (trial) four-factor interaction “was not significant, neither for spatial error, F(18, 576) = 0.44, p = 0.98, nor for initial cube speed, F(18, 576) = 0.48, p = 0.97” as we now report at the beginning of the Results section.

Page 16, lines 325-328: Shouldn’t this “quite successful” reparameterization be reflected in the comparison between trial 1 of that condition and trial 10 of the previously presented condition?

**Response :** This sentence was deleted from the new revision given that we now explain that the analysis on block order is performed in a separated section, and not on average performance anymore.

It is also not clear why the absence of motor adaptation speaks in favor of a reparameterization. Perhaps the authors need to be more explicit in what exactly they mean with ‘reparameterization’. But that there is no motor adaptation, at least the way that the authors compute it, does not necessarily mean that participants did the task successfully…the shown error of ~8% is something like 2 and 4 cm for the 25 and 50 cm conditions, which in my understanding this should be visually evident to the participants (unless the authors can show that the Weber’s fraction of such error perception is similar or larger than the errors reported here).

Such errors in trial 1 may also *not* be below the alpha level, and thus performance is considered ‘quite successful’ by the authors, either because the alpha level is set too low (due to the several comparisons planned by the authors) or due to variability across subjects (which is not shown and should be), or due to inappropriate testing (distribution of data).

**Response :** Regarding the latter comments about interindividual variability and data distribution, see our response before. About the fact that “the absence of motor adaptation speaks in favor of a reparameterization.” and “that there is no motor adaptation, at least the way that the authors compute it, does not necessarily mean that participants did the task successfully”, we now deleted most of this paragraph including this sentence the reviewer is referring to : “The results in the initial trial of the +10° condition suggest a quite successful reparameterization of the striking movement for the Balsa wood surface (see Fig 3), as illustrated by the absence of motor adaptation across trials and an initial spatial error that did not differ from 0, t(32) = 2.65, p > 0.05/12 = 0.004.”

Telling from the figures, one may even conclude that participants optimize energetic than accuracy factors. For instance, since the speed seems rather constant (most curves in Figure 3), it might be that people prefer to apply the same energy with their movement which leads to different initial cube speeds (depending on the material) and thus on different spatial errors. Obviously, this is not always the case, as nicely shown for the 0-deg condition, but can be an additional explanation of the rather variable spatial errors in several cases.

**Response :** As we put it in the Discussion, due to motor noise, there is variability in performance, even though it tends to improve across trials. The fact that in the last trial performance does not always reach 0% spatial error is partly due to motor noise. The reviewer incriminates the low alpha level due to multiple comparisons. However, although in the new revision, we increased the alpha level for the multiple comparisons in Fig 3 and 4, it did not change the conclusions. When the error at the last trial is significantly different from 0% we discuss this finding.

About the interpretation that “participants optimize energetic than accuracy factors [...] since the speed seems rather constant (most curves in Figure 3)”, let’s remember that this is average data. In contrast, Figs 5 and 6 show that cube speed varied much more, in the +10° condition, for example.

Page 16, lines 342-343: Please remind the reader about the purpose of this analysis. Why is the slope removed from the factors?

**Response :** We performed an omnibus ANOVA, and “we focused our analysis on interactions involving the trial factor.” (see beginning of Results section). In the revision, we now start by indicating that “The fourth-factor interaction between surface material, slope, target distance, and trial was not significant”. Then, the next interaction involving the trial factor was “three-factor interaction between surface material, slope and trial“ (see Fig 3). So, Fig 4 is simply the next significant interaction involving the trial factor that needs to be described.

What is the additional information compared to the previous analysis?

**Response :** This interaction (Fig 4) included the *Target distance* factor, in contrast to the previous one.

Page 18, line 387: What are the 24 tests that the authors imply here?

**Response :** In the new version we removed “instead of p < 0.05/24”.

And what are the four tests implied in line 390 of the same page? It is quite hard to follow.

**Response :** In the new version we added : “the four comparisons corresponding to the transition between the first and second block, and between the second and third block, for each level of Target distance”.

Page 21, line 443: “appropriate movement re-parameterization”. I still do not get this. Could you please be explicit and show which results and tests you refer to? Do you compare trials 1 and 10, or trial 1 against zero (or something else)?

**Response :** We now better explain that : ”Fig 7 illustrates the change in performance between the last trial of block 6 (last trial of the 25 cm blocks) and the first trial of block 7 (first trial of the 50 cm blocks). We used Bonferroni corrected (*p* < 0.05/4) t tests to examine if there was a significant performance change between those two trials (illustrated by arrows in Fig 7), and a significant spatial error with respect to zero (illustrated by asterisks in Fig 7). We theorized that a performance that not differing significantly from zero after a change in three physical properties of the stimulus would reflect adequate movement re-parameterization (in order to impart optimal initial cube speed).”

**Minor**

Please state explicitly the abbreviation of the International Society for Biomechanics (line 122, page 6).

**Response :** We modified the text accordingly.

Page 9, line 178: How is the cube’s movement onset defined?

**Response :** To determine the cube’s movement onset, the program looked for the maximum cube speed and then used a reverse while loop until the speed was smaller than 0.04 m/s. Accordingly, we added the following information in the revised manuscript : ”Cube’s movement onset was defined as a cube speed greater than 0.04 m/s.“

Page 9, equation 1: What does the ‘min’ as a nominator represent? The minimum of what? This formula is confusing. Also, why do the authors use 40% as their threshold when using the MSI for deceleration ?

**Response :** *min* applies to	$\left\{ t \left( \frac{Acceleration}{Min Acceleration} \right)> 0.4 \right\}$	in equation (1).

We rewrote the corresponding text as

Accordingly, the contact time point (tcontact) was determined for the [ tmaxFS ; tminCS ] interval as:

***t*_contact =_** $\boldsymbol{min}\left\{ \boldsymbol{t}\left( \frac{\mathrm{Acceleration}}{Min Acceleration} \right)> 0.4 \right\}-d\boldsymbol{t}$	(1)

Moreover to make this point clearer, we now explain in the revised manuscript that : “We used this criterion in order to avoid false detection of finger-cube contact, because after maximum finger speed (beginning of the time interval of interest) there was sometimes a slight deceleration (less than 40%) in finger movement before contact.”

Here, we also provide to the reviewer the Figure below so that he/she can visualize in the bottom panel what we mean by “slight deceleration (less than 40%) in finger movement before contact.”.

Page 12, equation 5: a parenthesis is redundant after θwrist?

**Response :** In the new revision this redundant parenthesis was deleted (now Equation 6).

Page 19, lines 405-406: Do the authors mean that the speed needs to increase that much to achieve perfect performance? Please clarify.

**Response :** We now clarify in the new revision that :” [...] optimal cube speed values (see Table 2) show that, when changing from -10° to +10° for the 50 cm target distance, one needs to increase cube speed by about 50% in the balsa wood condition (i.e., from 1.68 m/s to 2.50 m/s) and by about 70% in the aluminum condition (i.e., from 1.37 m/s to 2.30 m/s).”

Page 19, lines 407-408: I am sorry but I cannot follow this argument. Could you please clarify?

**Response :** Now, in the “Performance variables” sub-section of “Materials and Methods”, we mention the type of evidence in favor of movement re-parameterization based on subjective µ (internal model of friction), among which : “[...] traces of a generalization of the internal model of physics (including subjective µ) from one block to the next could also manifest in a greater learning speed in the new block.” This was a previous suggestion of Reviewer 1 : “one might attempt to extract some hints of possible internal model of friction existence, if learning speed would be compared between the first block of conditions and the second when surface friction was changed.”

In the new revised manuscript, we added “(see “Performance variables” section)” to remind the reader about the rationale we are using.

Page 29, line 643 (and elsewhere): Expressions such as “pretty good” are vague. How is “pretty good” defined? Please use objective language.

**Response :** The “pretty good” expression was introduced by McIntyre et al (2003) that we initially cited (see ref [28] in the Introduction : “Then, they would internalize this “pretty good” (i.e., functionally relevant although not perfect) internal model [28]”. We now added “ (i.e., functionally relevant, although not perfect)” in the Introduction and in the Discussion to define what is meant by “pretty good” internal model.

Yet, the authors state that spatial error was not different from zero, both in trial 1 and in trial 10. For this (and for several other comparisons), they do not report p-values in the text, but rather that the p-value is smaller than the set alpha level. Since the authors correct for multiple comparisons when looking at the spatial error in trial 1 of each of the 12 conditions, the set alpha is lower than the p-value. First, please report the p-values always, and remind the reader of the alpha level in the respective test.

**Response :** We now remind the reader of the alpha level when correcting for multiple comparisons, and provide the exact p-value except, as indicated in PLOS ONE guidelines, for *p* values less than 0.001 expressed as *p* < 0.001 (<https://journals.plos.org/plosone/s/submission-guidelines#loc-manuscript-organization>) : “P-values. Report exact p-values for all values greater than or equal to 0.001. P-values less than 0.001 may be expressed as p < 0.001, or as exponentials in studies of genetic associations.”
